# The Selective Rat Toxicant Norbormide Blocks K_ATP_ Channels in Smooth Muscle Cells But Not in Insulin-Secreting Cells

**DOI:** 10.3389/fphar.2019.00598

**Published:** 2019-05-23

**Authors:** Simona Saponara, Fabio Fusi, Ottavia Spiga, Alfonso Trezza, Brian Hopkins, Margaret A. Brimble, David Rennison, Sergio Bova

**Affiliations:** ^1^Department of Life Sciences, University of Siena, Siena, Italy; ^2^Department of Biotechnology, Chemistry and Pharmacy, University of Siena, Siena, Italy; ^3^Landcare Research, Lincoln, New Zealand; ^4^School of Chemical Sciences, University of Auckland, Auckland, New Zealand; ^5^Department of Pharmaceutical and Pharmacological Sciences, University of Padua, Padua, Italy

**Keywords:** norbormide, glibenclamide, KATP channel, patch-clamp, molecular modeling, docking

## Abstract

Norbormide is a toxicant selective for rats to which it induces a widespread vasoconstriction. In a recent paper, we hypothesized a role of ATP-sensitive potassium (K_ATP_) channels in norbormide-induced vasoconstriction. The current study was undertaken to verify this hypothesis by comparing the effects of norbormide with those of glibenclamide, a known K_ATP_ channel blocker. The whole-cell patch-clamp method was used to record K_ATP_ currents in myocytes freshly isolated from the rat and mouse caudal artery and from the rat gastric fundus, as well as in insulin-secreting pancreatic beta cells (INS-1 cells). Smooth muscle contractile function was assessed on either rat caudal artery rings or gastric fundus strips. Molecular modeling and docking simulation to K_ATP_ channel proteins were investigated *in silico*. Both norbormide (a racemic mixture of endo and exo isomers) and glibenclamide inhibited K_ATP_ currents in rat and mouse caudal artery myocytes, as well as in gastric fundus smooth muscle cells. In rat INS-1 cells, only glibenclamide blocked K_ATP_ channels, whereas norbormide was ineffective. The inhibitory effect of norbormide in rat caudal artery myocytes was not stereo-specific as both the endo isomers (active as vasoconstrictor) and the exo isomers (inactive as vasoconstrictor) had similar inhibitory activity. In rat caudal artery rings, norbormide-induced contraction was partially reverted by the K_ATP_ channel opener pinacidil. Computational approaches indicated the SUR subunit of K_ATP_ channels as the binding site for norbormide. K_ATP_ channel inhibition may play a role in norbormide-induced vasoconstriction, but does not explain the species selectivity, tissue selectivity, and stereoselectivity of its constricting activity. The lack of effect in INS-1 cells suggests a potential selectivity of norbormide for smooth muscle K_ATP_ channels.

## Introduction

Norbormide, a selective rat toxicant, induces a lethal effect in rats but has little or no effect in non-rat species, including humans, and for this reason, it has been marketed for many years as an eco-sustainable pesticide (Roszkowski, 1965). The mechanism/s underlying the toxic effect of norbormide in rats is/are not understood, although existing evidence supports the idea that this toxicant induces a marked and irreversible vasoconstriction of rat peripheral vessels by targeting a receptor abundantly and/or selectively expressed in the rat peripheral artery myocytes (Bova et al., 2001). Norbormide contractile effect is endothelium-independent (Bova et al., 1996) and is restricted to rat vascular smooth muscle (Bova et al., 2003). Intriguingly, in rat conduit arteries (i.e., aorta), in rat non-vascular muscles, and in all muscles from non-rat species, norbormide, at concentrations partially overlapping those that induce vasoconstriction in rats, shows vasorelaxant properties, attributed to an inhibitory effect on Ca_V_1.2 channels (Bova et al., 2003).

From a chemical perspective, norbormide exists as a racemic mixture of up to eight stereoisomers, namely, four endo and four exo. Among them, only the endo isomers show rat-specific vasoconstrictor activity, being lethal to rats (Poos et al., 1966). Collectively, these observations indicate that the contractile activity of norbormide is not only species- and tissue-selective but also stereo-specific, thus suggesting the existence of a highly specific target for this compound in rat vessels.

K_ATP_ channels are a heterogeneous family of ion channels physiologically regulated by the intracellular concentration of ATP. They are composed of two subunits: the K_ir_ subunit, forming the channel pore, and the SUR subunit, which binds ATP and exerts a regulatory role over the K_ir_ subunit (Yokoshiki et al., 1998). Various isoforms of K_ir_ and SUR exist and assemble in differing partnerships to form tissue-specific K_ATP_ channels (Shi et al., 2005). In vascular smooth muscle, sarcolemmal K_ATP_ channels play a pivotal role in the regulation of vessel tone and blood pressure. In particular, activation of these channels allows K^+^ efflux from the cytoplasm to the extracellular space with a net loss of positive charges. This leads to membrane hyperpolarization, Ca_V_1.2 channel closure, cytoplasmic Ca^2+^ concentration decrease, and vasodilation (Ko et al., 2008; Foster and Coetzee, 2016). Conversely, K_ATP_ channel inhibition causes vasoconstriction. Compounds capable of activating K_ATP_ channels, e.g., pinacidil and cromakalim, are effective relaxant agents *in vitro* and show powerful hypotensive effects *in vivo* (Ashwood et al., 1986; Gollasch et al., 1995). In contrast, inhibition of vascular K_ATP_ channels contributes to the contractile response induced by receptor-coupled vasoconstrictor agents (Bonev and Nelson, 1996) regulating vascular tone, especially in resistance and coronary arteries (Tinker et al., 2014).

Glibenclamide is a sulfonylurea widely used as an antidiabetic agent (Rydén et al., 2014). In pancreatic beta cells, in fact, it blocks K_ATP_ channels, thus allowing Ca^2+^ to enter the cytoplasm and stimulate insulin secretion that, in turn, reduces blood glucose concentration (Ashcroft and Rorsman, 1989). Although glibenclamide displays high affinity for the pancreatic K_ATP_ channel isoform, it also affects K_ATP_ channels in other tissues and is therefore widely used *in vitro* as a pharmacological tool.

We have recently demonstrated that a fluorescent derivative of endo-norbormide (NRB-AF12) features a subcellular fluorescence profile similar to that of ER-Tracker^®^, a commercially available fluorescent derivative of glibenclamide (D’Amore et al., 2016; D’Amore et al., 2018; Forgiarini et al., 2019). This phenomenon occurred in several cell types, including freshly isolated rat caudal artery myocytes, where norbormide behaves as a contracting agent. Based on these results, we hypothesized that norbormide and glibenclamide can potentially bind to the same cellular target, namely, K_ATP_ channels, and that these channels can play a role in norbormide-induced vasoconstriction. This hypothesis was assessed in the present study by: i) studying norbormide effects on K_ATP_ currents recorded in vascular and non-vascular myocytes, as well as in INS-1 beta cells; ii) evaluating its functional activity on smooth muscle preparations; and iii) performing an *in silico* analysis to identify its potential binding site in K_ATP_ channels. In all these experiments, glibenclamide was used as a reference K_ATP_ inhibitor.

## Materials and Methods

All animal care and experimental protocols conformed to the European Union Guidelines for the Care and Use of Laboratory Animals (European Union Directive 2010/63/EU) and had been approved by the Italian Department of Health (666/2015-PR, 650/2015, 41451.N.ZRS/2018).

### Vasoconstriction Assay

Two-millimeter-long caudal artery rings were obtained from the main (ventral) caudal artery of male Wistar rats (160–230 g, Charles River Italia, Calco, Italy) anesthetized by CO_2_ and killed by decapitation.

The rings, cleaned of the adventitia, were mounted by inserting two 100-µm-thick tungsten wires into their lumen in home-made myographs, as previously described (Bova et al., 2003). The devices holding the rings were then immersed into 20-ml double-jacketed organ baths containing a salt solution of the following composition (in mM): NaCl 125, KCl 5, MgSO_4_ 1, KH_2_PO_4_ 1.2, CaCl_2_ 2.7, NaHCO_3_ 25, and glucose 11, pH 7.35, bubbled with a mixture of O_2_ (95%) and CO_2_ (5%), and maintained at 37°C. At the beginning of the experiment, a preload of 2 g (1 g/mm) was applied to each ring. The preloaded rings were left to equilibrate for at least 60 min and then repeatedly stimulated with 90 mM KCl and 10 µM phenylephrine until two consecutive reproducible contractile responses to each stimulus were obtained. The contractile force was measured through an isometric force transducer (2B Instruments, Milan, Italy) coupled to an analog-to-digital converter (PowerLab) and was displayed on the monitor of a PC. The experiments were conducted in rings mechanically deprived of the endothelium; the absence of a functional endothelium was confirmed by the lack of carbachol-induced relaxation of rings precontracted with phenylephrine.

### Rat Fundus Assay

Gastric fundus strips were obtained from the stomach of male Wistar rats (200–250 g, Charles River Italia, Calco, Italy) anesthetized (i.p). with a mixture of Zoletil 100^®^ (7.5 mg/kg tiletamine and 7.5 mg/kg zolazepam; Virbac Srl, Milano, Italy) and Xilor^®^ (4 mg/kg xylazine; Bayer, Milan, Italy) containing heparin (5,000 U/kg), decapitated, and bled. The stomach was removed, opened along the longitudinal axis of the greater curvature and washed in cold Ca^2+^-free physiological salt solution (Ca^2+^-free PSS) containing the following (in mM): NaCl 118, KCl 4.7, KH_2_PO_4_ 1.2, MgCl_2_ 1.2, NaHCO_3_ 25, and glucose 11.5. Smooth muscle strips (1–2 mm in width; 1.5–2 cm in length), dissected from the circular layer of the anterior fundus wall, were transferred into 25-ml organ bath chambers filled with Ca^2+^-free PSS, pH 7.4, bubbled with O_2_ (95%) and CO_2_ (5%) gas mixture, and maintained at 37°C. Strips, connected to isometric transducers (BLPR, WPI, Berlin, Germany), were stretched to a tension of 1 g. After equilibration for 15 min, 2.5 mM Ca^2+^ was added and the strips were allowed to develop stable, spontaneous tone over a 30-min period. Afterward, they were challenged with two consecutive stimulations with 60 mM K^+^ (K60), until a stable response was obtained. After 45 min of washing in PSS, either norbormide or glibenclamide was added and left in contact with tissues for 15 min, followed by a K60 stimulation to test for muscle functional integrity. Tension was expressed as a percentage of the initial response to K60, which was regarded as 100% (Fusi et al., 1998).

### Electrophysiological Experiments

#### Cell Isolation Procedure From Rat and Mouse Tail Main Artery

Rat tail artery myocytes were obtained from the same rats used for the fundus assay (see above); mice tail artery myocytes were obtained from C57BL6 mice (20–25 g, Internal breeding of the Department of Pharmaceutical and Pharmacological Sciences of the University of Padua) killed by cervical dislocation. The tail artery was cut, cleaned of skin, and placed in external solution (containing in mM: 130 NaCl, 5.6 KCl, 10 4-(2-hydroxyethyl)-1-piperazineethanesulfonic acid (HEPES), 20 glucose, 1.2 MgCl_2_, and 5 Na-pyruvate; pH was adjusted to 7.4 with NaOH). Smooth muscle cells were freshly isolated from two mice arteries and a 5-mm-long piece of rat artery by incubation at 37°C for 40–45 min in 2 ml of 0.1 mM Ca^2+^ external solution containing 20 mM taurine (prepared by replacing NaCl with equimolar taurine), 1.25 mg/ml collagenase (type XI), 1 mg/ml soybean trypsin inhibitor, and 1 mg/ml bovine serum albumin. This solution was gently bubbled with an O_2_ (95%) and CO_2_ (5%) gas mixture to stir the enzyme solution, as previously described (Fusi et al., 2016). Cells were stored in 0.05 mM Ca^2+^ external solution containing 20 mM taurine and 0.5 mg/ml bovine serum albumin at 4°C under normal atmosphere, and were used within 2 days of isolation (Mugnai et al., 2014).

#### Cell Isolation Procedure From Rat Gastric Fundus

Gastric smooth muscle cells were freshly isolated from rat gastric fundus strips obtained from the same rats used for the fundus assay (see above). The strips were digested for 40–45 min at 37°C in 2 ml of nitrate-rich digestion solution (in mM: NaCl 55, NaNO_3_ 65, KCl 5, Na-pyruvate 5, glucose 10, taurine 10, HEPES 10, and MgCl_2_ 1.2; pH 7.4) containing 2 mg collagenase (type I), 2.5 mg bovine serum albumin, and 3 mg soybean trypsin inhibitor, bubbled with an O_2_ (95%) and CO_2_ (5%) gas mixture (Fusi et al., 2001).

Thereafter, cells were mechanically dispersed with a plastic pipette in a modified Kraft-bruhe (KB) solution [containing 1 mg bovine serum albumine and (in mM) NaCl 105, KH_2_PO_4_ 7, KCl 5, glucose 5, taurine 10, HEPES 10, MgCl_2_ 1.6, Na-pyruvate 2.5, creatine 1.7, oxalacetate 2, Na_2_ATP 1.5, and ethylene glycol-bis(2-aminoethylether)-N,N,N´,N´-tetraacetic acid (EGTA) 0.1; pH adjusted to 7.25 with NaOH] and used for experiments within 10 h of isolation. During this time, they were stored at 4°C in KB solution containing bovine serum albumin.

#### INS-1 Cells

INS-1 beta cells (Asfari et al., 1992), kindly provided by Dr. Andrea Venerando (Department of Comparative Biomedicine and Food Science, University of Padua, Italy), were grown in RPMI 1640 medium containing 11.1 mM D-glucose, 1 mM sodium pyruvate, 50 μM β-mercaptoethanol, 2 mM glutamine, 10 mM HEPES, 10% fetal calf serum (FCS), 100 U/ml penicillin, 100 μg/ml streptomycin, and 250 ng/ml amphotericin B at 37°C in humidified 5% CO_2_ and 95% air (Kittl et al., 2016). Cell cultures were passaged once a week using the standard trypsin/EDTA treatment.

#### Whole-Cell Patch-Clamp Recordings

Cells were continuously superfused with recording solution using a peristaltic pump (LKB 2132, Bromma, Sweden) at a flow rate of 400 μl/min.

The conventional whole-cell patch-clamp method was applied to voltage-clamp smooth muscle cells (Saponara et al., 2011). Recording electrodes were pulled from borosilicate glass capillaries (WPI, Berlin, Germany) and fire-polished to obtain a pipette resistance of 2–5 MΩ when filled with internal solution.

An Axopatch 200B patch-clamp amplifier (Molecular Devices Corporation, Sunnyvale, CA, USA) was used to generate and apply voltage pulses to the clamped cells and to record the corresponding membrane currents. At the beginning of each experiment, the junction potential between the pipette and bath solution was electronically adjusted to zero. Current signals, after compensation for whole-cell capacitance and series resistance (between 70% and 75%), were low-pass filtered at 2 kHz and digitized at 3 kHz prior to being stored on the computer hard disk. Electrophysiological responses were tested at room temperature (20–22°C).

The osmolarity of the external solution and that of the internal solution were measured with an osmometer (Osmostat OM 6020, Menarini Diagnostics, Florence, Italy) (Fusi et al., 2005).

Analysis of data was accomplished by using pClamp 9.2.1.8 software (Molecular Devices Corporation).

#### K_ATP_ Current Recording in Tail Artery and Gastric Myocytes

Recording solution contained the following (in mM): NaCl 25, KCl 140, HEPES 10, glucose 10, MgCl_2_ 1, CaCl_2_ 0.1, and tetraethylammonium (TEA) 1; pH was adjusted to 7.4 with NaOH (320 mosmol). Pipette solution consisted of the following (in mM): KCl 140, HEPES 10, EGTA 10, MgCl_2_ 1, glucose 5, Na_2_ATP 0.1, KADP 1, and Na_2_GTP 0.1; pH was adjusted to 7.3 with KOH (290 mosmol). To minimize voltage-dependent K^+^ currents, K_ATP_ currents were recorded at a steady membrane potential (*V*_h_) of −50 mV using a continuous gap-free acquisition protocol. Currents, activated by the K_ATP_ channel opener pinacidil (10 µM), did not run down during the following 10 min under these conditions (data not shown). Care was taken to complete each experiment within this period. Current values were corrected for leakage using 10 µM glibenclamide, which completely blocked K_ATP_ currents (Trezza et al., 2018).

Ca^2+^ is known to stabilize the cell membrane. Due to its low concentration in the recording solution, the seal between the pipette tip and the cell membrane was frequently lost a few minutes after breaking the patch to get the whole-cell configuration. Thus, only single concentrations of norbormide were assessed in each myocyte. Furthermore, as few cells did not respond to pinacidil stimulation, norbormide was always added after pinacidil had induced a stable current amplitude.

#### K_ATP_ Current Recording in INS-1 Cells

Recording solution contained the following (in mM): NaCl 140, KCl 5.6, CaCl_2_ 2.5, MgCl_2_ 1.5, HEPES 10, D-glucose 4.5, and mannitol 5; pH was adjusted to 7.4 with NaOH (300 mosmol). Pipette solution contained the following (in mM): potassium D-gluconate 120, NaCl 5, KCl 10, CaCl_2_ 2, MgCl_2_ 4, Mg_2_ATP 2, HEPES 5, EGTA 10, and raffinose 5; pH was adjusted to 7.2 with KOH.

K_ATP_ currents were recorded by means of an “activator-free” protocol, consisting in 500-ms pulses to −80 and −60 mV, delivered at 10-s intervals, from a *V*_h_ of −70 mV, as previously described (Kittl et al., 2016). Under these conditions, currents measured were almost entirely K_ATP_ currents, as proved by the 10 µM glibenclamide blockade. The latter was used to correct current values for leakage.

### Chemicals

Endo- and exo-norbormide isomers were prepared at the University of Auckland, New Zealand. Endo-norbormide was synthesized as previously described (Brimble et al., 2004). The exo isomer was obtained from a solution of endo-norbormide (1.00 g, 1.95 mmol) in mixed xylenes (20 ml) heated at 140°C for 30 h, and the solvent was then removed *in vacuo*. Purification by flash chromatography (petroleum ether/ethyl acetate, 1:1) afforded exo-norbormide as a mixture of stereoisomers (R/S/T/X) (beige solid; 0.80 g, 1.56 mmol, 80%). ^1^H NMR (400 MHz, CDCl3) δ 2.89 (0.15H, dd, *J* = 7.3, 0.9 Hz, S/H-2), 2.92–2.95 (0.6H, m, T/H-2, X/H-2), 2.97 (0.25H, dd, *J* = 7.3, 0.9 Hz, R/H-2), 3.03 (0.45H, dd, *J* = 7.3, 1.1 Hz, X/H-3), 3.06 (0.15H, dd, *J* = 7.3, 1.1 Hz, S/H-3), 3.25 (0.15H, dd, *J* = 7.3, 1.1 Hz, T/H-3), 3.41 (0.25H, dd, *J* = 7.3, 1.1 Hz, R/H-3), 3.49–3.50 (0.15H, m, S/H-4), 3.73–3.74 (0.25H, m, R/H-1), 3.76–3.77 (0.6H, m, X/H-1, T/H-4), 3.95–3.96 (0.45H, m, X/H-4), 4.09–4.10 (0.25H, m, R/H-4), 4.33–4.35 (0.3H, m, S/H-1, T/H-1), 5.74–5.76 (0.4H, m, T/H-6, R/H-6), 5.80 (0.15H, s, S/OH), 5.86 (0.45H, s, X/OH), 6.15 (0.45H, dd, *J* = 3.4, 1.1 Hz, X/H-6), 6.18 (0.15H, s, T/OH), 6.19 (0.15H, dd, *J* = 3.4, 1.1 Hz, S/H-6), 6.51 (0.25H, s, R/OH), 6.85–7.65 (16H, m, Ar), 8.42–8.59 (2H, m, αPyr). RP-HPLC: *t_R_* = 45.7 (S), 51.7 (T), 58.7 (X), 61.1 (R) min (purity λ_254nm_ = >99%). A ^1^H NMR spectrum and RP-HPLC chromatogram are reported in **Supplementary Figure 1**.

Collagenase (types I and XI), trypsin inhibitor, bovine serum albumin, TEA, EGTA, HEPES, taurine, pinacidil, glibenclamide, RPMI 1640, β-mercaptoethanol, glutamine, FCS, penicillin, streptomycin, and amphotericin B were resourced from Sigma Chimica (Milan, Italy). Norbormide (mixed endo and exo isomers) was a kind gift from I.N.D.I.A. Industrie Chimiche Srl, Padua, Italy. Endo- and exo-norbormide were synthesized at the School of Chemical Sciences, University of Auckland, New Zealand. Norbormide, pinacidil, and glibenclamide, dissolved directly in dimethyl sulfoxide (DMSO), were diluted at least 1,000 times prior to use. All solutions were stored at −20°C and protected from light by wrapping containers with aluminum foil. The resulting concentrations of DMSO (below 0.1%, v/v) had no influence on tissue or cell responses.

### Statistical Analysis

Individual values, contributing to calculation of the groups’ mean ± SEM values, derived from independent cells, rings, or strips that, sometimes, were isolated from the same animal. To ensure that mean values are representative of the population, however, cells or rings included in the same group were isolated from at least three different animals.

Statistical analysis and significance, as measured by either repeated measures ANOVA (followed by Dunnett *post hoc* test) or Student’s *t* test for paired samples (two-tailed), were obtained using GraphPad Prism version 5.04 (GraphPad Software Inc., San Diego, CA, USA). *Post hoc* tests were performed only when ANOVA found a significant value of *F* and no variance in homogeneity. In all comparisons, *P* < 0.05 was considered significant.

### Homology Model Building and Validation

The cryo-electron microscopy (cryo-EM) structure of the pancreatic β-cell K_ATP_ channel bound to ATP and glibenclamide, with Protein Data Bank identity (PDB) ID 6BAA (Martin et al., 2017), was used as the main template for the homology model (Protein Model Portal; Haas et al., 2013). The UniProt database (www.uniprot.org) sequences of *Rattus norvegicus* K_ATP_ channel characterized by K_ir_6.1 Q63664 and K_ir_6.2 P70673 subunits, together with the SUR1 Q09429 and SUR2 Q63563 subunits, obtained with ClustalX tool (Jeanmougin et al., 1998), were used for alignment. PyMOL plugin PyMod 2.0 (Janson et al., 2017) was used to achieve a protein structure prediction by means of ClustalO (Sievers et al., 2011). Sequence alignment between *R. norvegicus* K_ir_6.1 and K_ir_6.2 exhibited 82% query coverage and 74% identity with 0.0 *E* value, while that between *R. norvegicus* SUR1 and SUR2 showed a query coverage of 98% with an identity of 72.8% with 0.0 *E* value.

**Figure 1:**
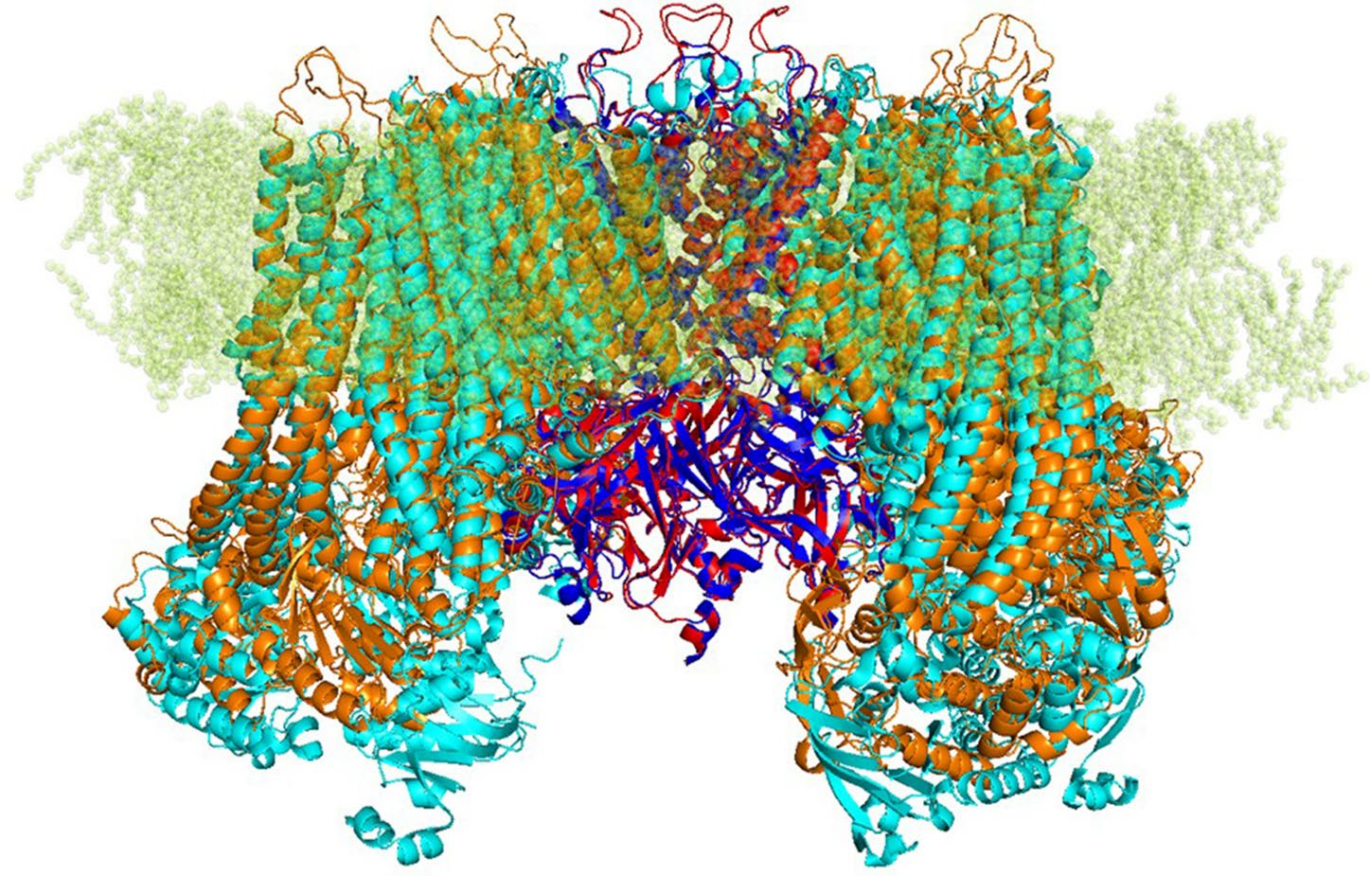
Strucutre of transmembrane K_ATP_ channels. Ribbon representation of superimposed K_ir_6.1 homology model (red) and K_ir_6.2 cryo-EM structure 6BAA (blue) complexed with SUR subunits, SUR2 homology model (orange), and SUR1 cryo-EM structure 6BAA (cyan).

Based on those alignments, 3D models for target protein were generated by using Modeller integrated in Pymod 2.0. The quality of the modeled subunits (K_ir_6.1 and SUR2) was assessed using local factors such as packing quality, backbone conformation, bond length, and side-chain planarity. Structural superimposition between the K_ir_6.1 and SUR2 model with the cryo-EM structure of K_ir_6.2 and SUR1 (PDB ID 6BAA), as reported in **Figure 1**, showed a high structural similarity with backbone root-mean-square distance (RMSD) values of 0.57 and 1 Å, respectively. Structure validation indicated that the protein homology models of subunits K_ir_6.1 and SUR2 possessed reasonable 3D structure with a good stereochemical quality of 98.8% and 97% of the amino acid residues in the most favored regions, respectively, as assessed by Ramachandran plot. Energy minimization protocol in the simple point charge water model and the RMSD were computed for the K_ATP_ channel K_ir_6.1/SUR2 three-dimensional model by using GROMACS 2016.4 software package (Abraham et al., 2015).

### Molecular Docking

The molecular structure of norbormide (10468605 ChemSpider ID) was acquired through ChemSpider in Mol format (www.chemspider.com). The norbormide and glibenclamide pdbqt format was generated by using Open Babel tools, adding Gasteiger charge (O’Boyle et al., 2011), whereas the pdbqt format of the proteins was generated using utility scripts included in the AutoDock tools graphical user interface. Norbormide was subjected to the steepest descent minimization (500 steps at 0.02-Å step size) to remove unfavorable clashes, followed by 100 steps of conjugate gradient minimization (0.02-Å step size; Pettersen et al., 2004). Docking simulation studies of ligands against the K_ir_6.1/SUR2 model and the K_ir_6.2/SUR1 cryo-EM structure (Martin et al., 2017) were performed using the flexible side-chain protocol based on Iterated Local Search Global Optimizer Algorithm of AutoDock/VinaXB (Koebel et al., 2016). Protein–ligand network interaction was evaluated with protein–ligand interaction profiler (PLIP) (Salentin et al., 2015). PyMOL 1.7.6.0 was used as the molecular graphics system (The PyMOL Molecular Graphics System, Version 1.8; Schrödinger, LLC, New York, NY, USA).

## Results

### Effects of Norbormide on K_ATP_ Currents in Vascular and Non-Vascular Single Cells

**Figure 2:**
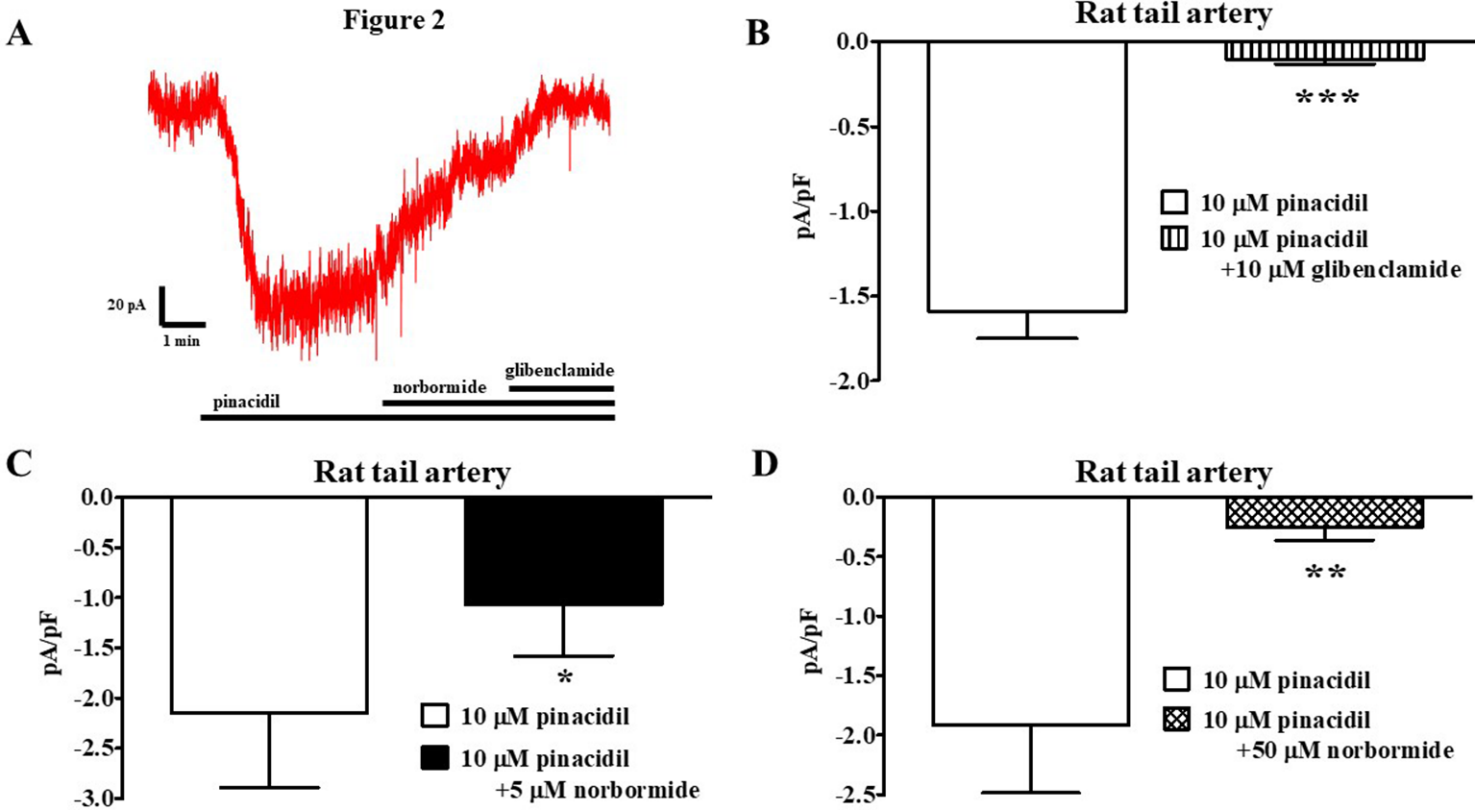
Effect of norbormide on K_ATP_ currents of isolated rat tail artery myocytes. **(A)** Representative whole-cell recordings of inward currents elicited by 10 µM pinacidil at a *V*_h_ of −50 mV. The effect of 5 µM norbormide as well as of 10 µM glibenclamide is shown. **(B)** Pinacidil (10 µM) activated glibenclamide-sensitive K_ATP_ currents, which were inhibited by either **(C)** 5 µM or **(D)** 50 µM norbormide. Columns are mean ± SEM (*n* = 5–7). *P < 0.05, **P < 0.01, ***P < 0.001, Student’s *t* test for paired samples.

**Figure 3:**
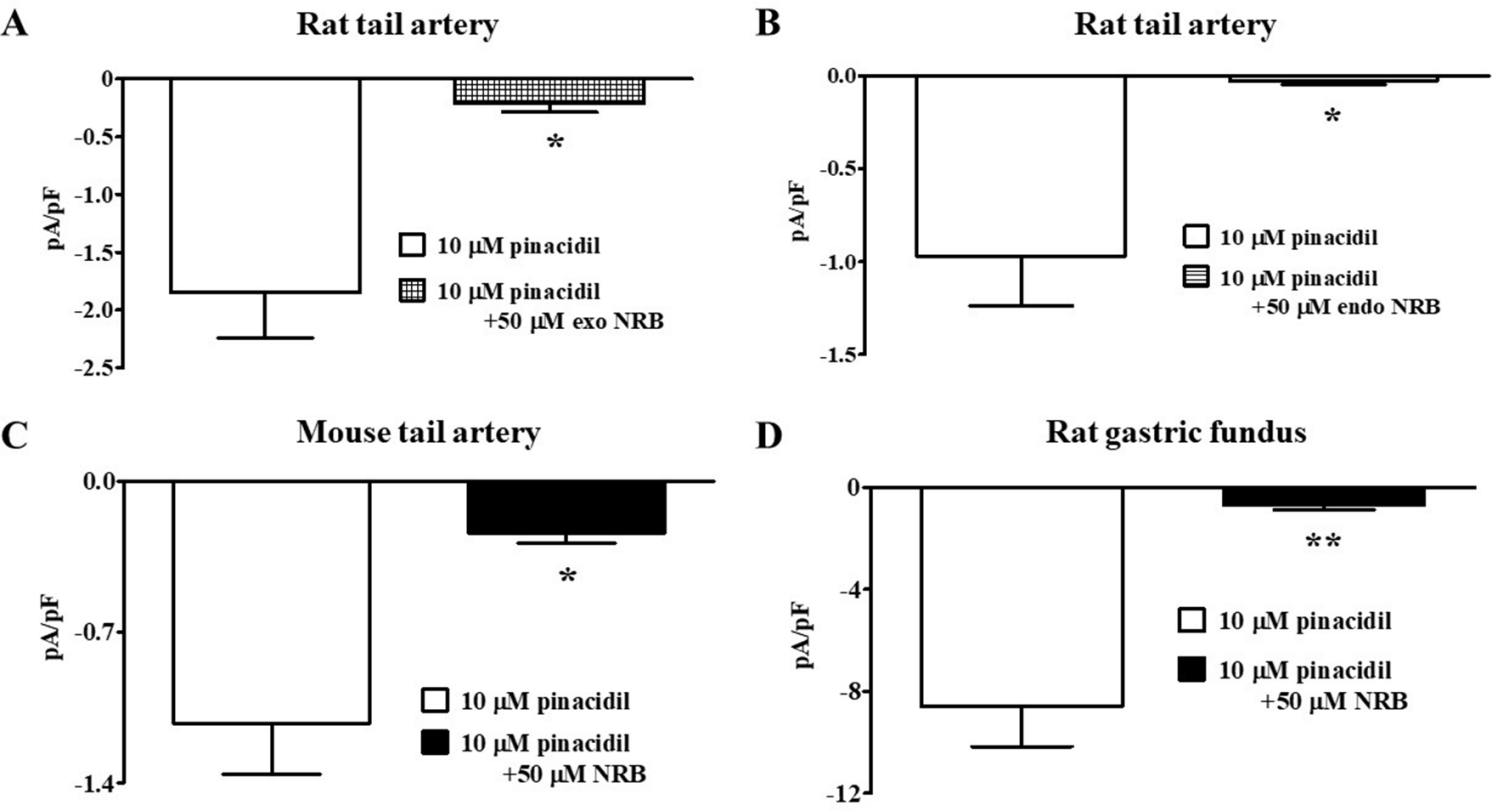
Effect of norbormide on K_ATP_ currents of isolated rat and mouse tail artery or rat fundus myocytes. **(A** and **B)** Pinacidil (10 µM) activated glibenclamide-sensitive rat tail artery myocytes K_ATP_ currents, which were inhibited by either **(A)** 50 µM exo-norbormide (NRB) or **(B)** 50 µM endo NRB. **(C** and **D)** Pinacidil (10 µM) activated glibenclamide-sensitive **(C)** mouse tail artery and **(D)** rat gastric fundus myocytes K_ATP_ currents, which were inhibited by 50 µM NRB. Columns are mean ± SEM (*n* = 4–5). *P < 0.05, **P < 0.01, Student’s *t* test for paired samples.

The effect of norbormide on K_ATP_ channels was assessed in vascular (myocytes freshly isolated from rat and mouse tail main artery) and non-vascular (myocytes freshly isolated from rat gastric fundus) single cells and single INS-1 cells. To limit activation of K_V_ and K_Ca_1.1 channels in the vascular and gastric myocytes, K_ATP_ currents were induced at a *V*_h_ of −50 mV in the presence of 0.1 mM ATP and 1 mM ADP in the pipette solution, and 1 mM TEA in the recording solution. In rat vascular myocytes, the K_ATP_ channel opener pinacidil (10 µM) activated an inward current that was significantly antagonized by the K_ATP_ channel blocker glibenclamide (10 µM; **Figure 2A and B**). Racemic norbormide (5 and 50 µM) inhibited, in a concentration-dependent manner, glibenclamide-sensitive currents recorded in the presence of pinacidil (**Figure 2C and D**). Of interest, we found that exo-norbormide (50 µM) and endo-norbormide (50 µM) had an inhibitory efficacy that was comparable to that of norbormide (50 µM) (**Figure 3A and B**). Norbormide (50 µM) also caused a significant reduction of K_ATP_ current in mice vascular (**Figure 3C**) and in rat gastric fundus myocytes (**Figure 3D**), similarly to that observed in rat vascular myocytes.

**Figure 4:**
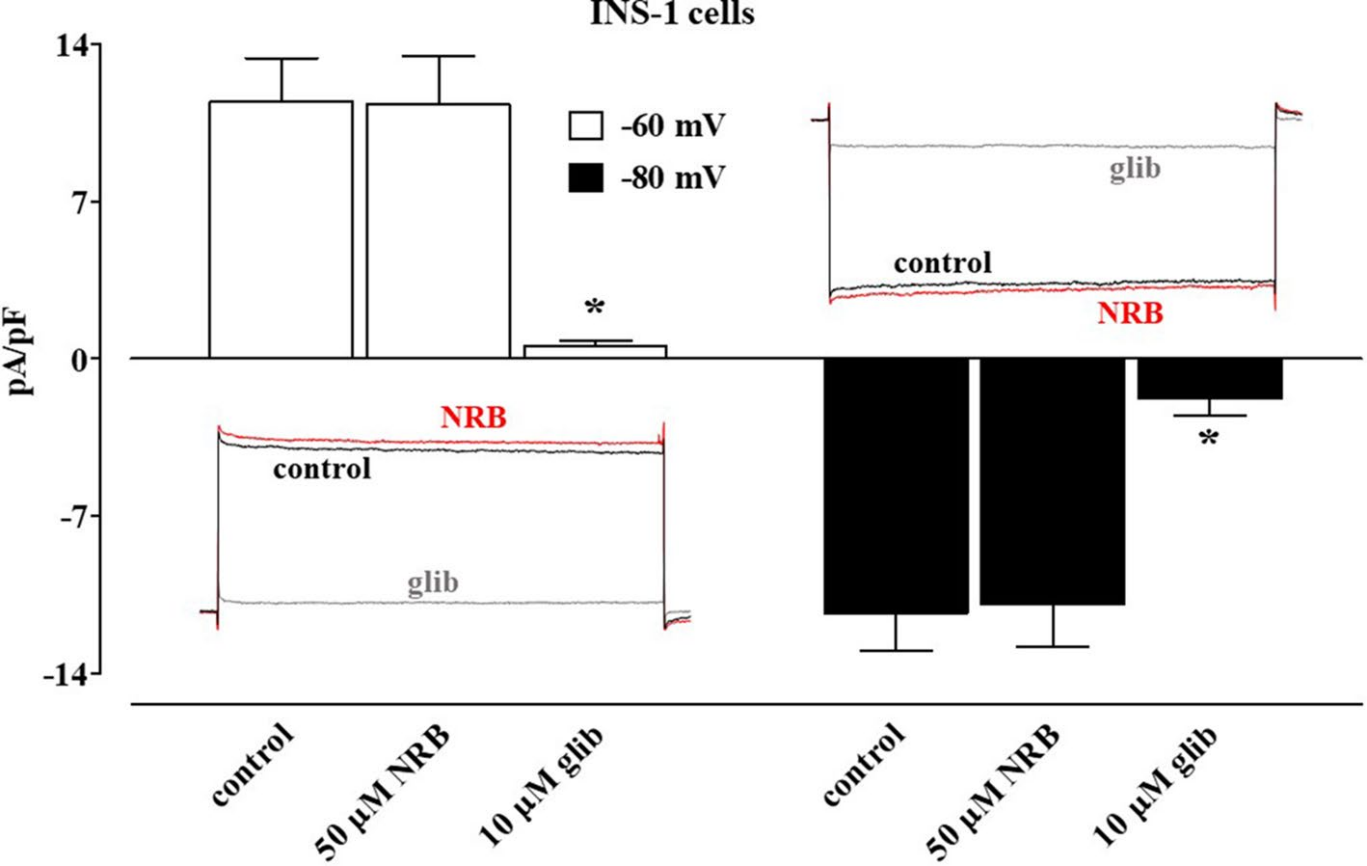
Effect of norbormide on K_ATP_ currents of INS-1 cell. K_ATP_ currents were recorded at a *V*_h_ of −70 mV during 500-ms pulses to −60 mV (white columns) and −80 mV (black columns) in the absence (control) or presence of 50 µM norbormide (NRB) as well as NRB plus 10 µM glibenclamide (glib). Columns are mean ± SEM (*n* = 7). Insets: average traces of original K_ATP_ currents, recorded from seven cells, during 500-ms pulses to −60 mV (left) or −80 mV (glib) in the absence (control) or presence of NRB as well as NRB plus glibenclamide. *P* < 0.001, ANOVA and Dunnett post test.

In direct contrast, norbormide (50 µM) did not affect K_ATP_ current recorded in INS-1 cells, which was significantly inhibited by glibenclamide (10 µM; **Figure 4**).

### Effect of Norbormide and Glibenclamide on the Mechanical Activity of Rat Caudal Artery Rings and Fundus Strips

These experiments were undertaken to assess and compare the mechanical effects of norbormide and glibenclamide on rat caudal artery and fundus preparations.

**Figure 5:**
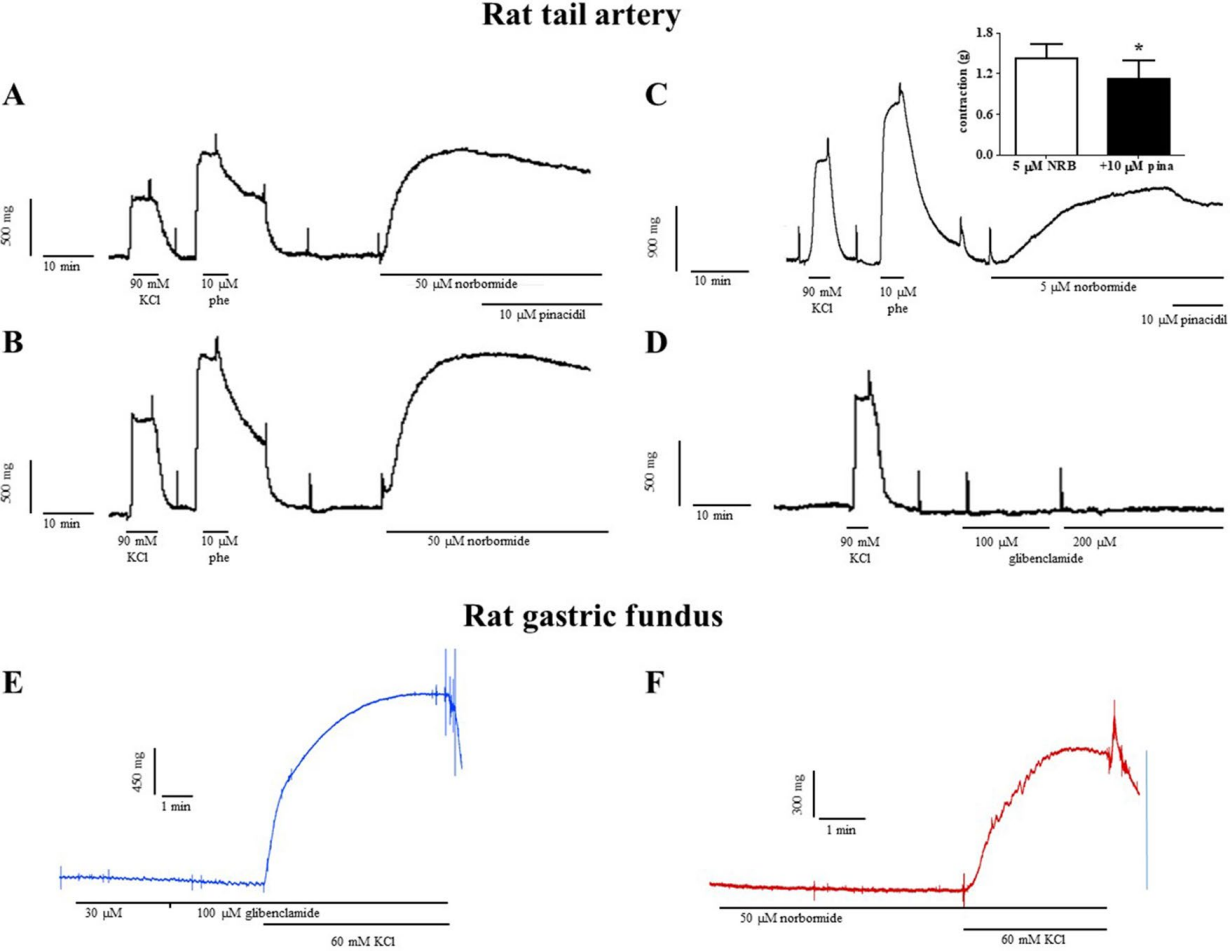
Effects of norbormide and glibenclamide on the resting tone of rat caudal artery rings and gastric fundus strips. **(A–D)** Rat caudal artery rings. **(A)** Original recording showing the contractile effect of a maximal concentration of norbormide (50 µM). The addition of 10 µM pinacidil did not affect active muscle tone (compared to panel **B**). **(B)** Control experiment, performed in parallel with that represented in **(A)**, showing that norbormide-induced contraction spontaneously relaxes after the plateau phase, thus suggesting that the relaxation observed in **(A)** is not pinacidil-dependent. **(C)** Original recording showing the contractile effect of a low concentration of norbormide (5 µM). The addition of 10 µM pinacidil caused a partial relaxation of the active muscle tone. Inset: effect of pinacidil on rat caudal rings precontracted with 5 µM norbormide. On the ordinate scale, contraction is reported in g (*n* = 6; *P < 0.05, Student’s *t* test for paired samples). **(D)** Glibenclamide, up to 200 µM, was unable to affect ring resting tone. **(A–D)** The contraction induced by 90 mM KCl and/or 10 µM phenylephrine is also shown. Neither **(E)** glibenclamide nor **(F)** norbormide contracted rat gastric fundus strips. The effect of 60 mM KCl is also shown. Traces are representative of five (norbormide) or six (glibenclamide) similar experiments.

In vascular rings, norbormide, at the same concentration (50 µM) that almost completely blocked K_ATP_ channels in rat caudal artery myocytes, produced a contractile response that was 140% of that induced by 90 mM KCl (**Figure 5A and B**). Norbormide-induced contraction was not affected by the same concentration of pinacidil (10 µM) used to activate K_ATP_ channels in patch-clamp experiments. However, when norbormide concentration was lowered to 5 µM, the addition of pinacidil indeed caused a 20% relaxation of the active tone (**Figure 5C**). Glibenclamide, up to 200 µM, did not modify basal tension in rat caudal artery rings (**Figure 5D**).

Fifty micromolar NRB-induced vasoconstriction was reverted upon washout in about 90 min (data not shown). Furthermore, the following addition of 10 µM phenylephrine or 90 mM KCl elicited contractile responses comparable to those recorded before NRB challenging (data not shown).

Both norbormide (50 µM) and glibenclamide (up to 100 µM) were unable to contract rat gastric fundus strips (**Figure 5E and F**). The subsequent addition of 60 mM KCl, however, elicited a contraction that measured 67.2 ± 10.8% (*n* = 5) and 90.5 ± 3.5% (*n* = 6) of that recorded in the absence of the drug, respectively.

### Molecular Modeling and Docking Simulation

**Figure 6:**
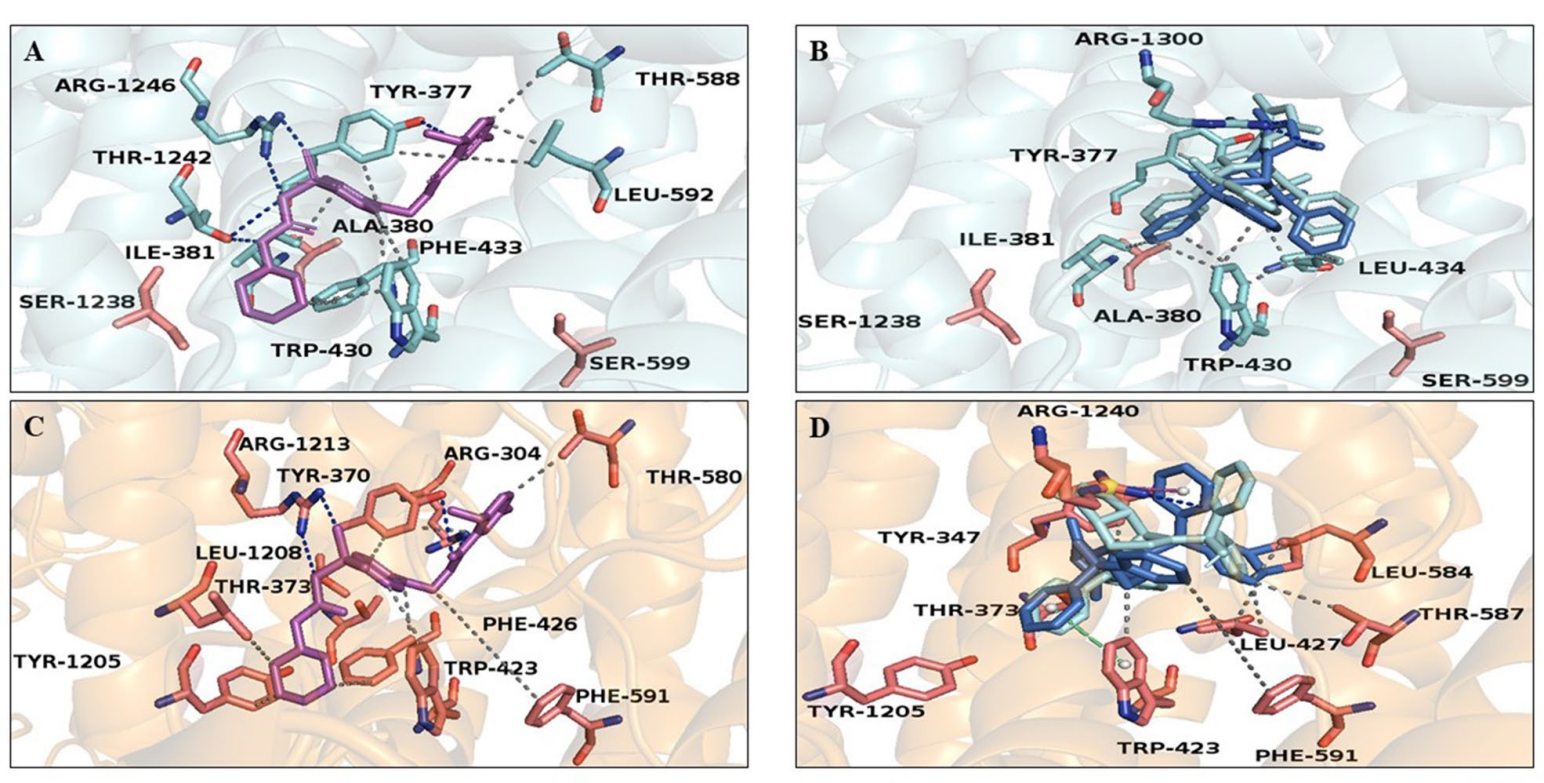
Glibenclamide and norbormide binding site. **(A** and **B)** Inhibited SUR2 cartoon structure (PDB accession number 6BAA) showing only the interactions between residues’ binding site (cyan sticks) and **(A)** glibenclamide (purple sticks) or **(B)** norbormide (blue sticks). **(C** and **D)** SUR1 cartoon model structure, showing only the interactions between residues’ binding site (orange sticks) and **(C)** glibenclamide (purple sticks) or **(D)** norbormide (blue sticks).

The predicted K_ATP_ 3D model was comparable to the 6BAA template (Martin et al., 2017). Norbormide (both endo and exo conformation) was found to be flexible and free to bind the two K_ATP_ channels, namely, K_ir_6.1/SUR2 in smooth muscle cells and K_ir_6.2/SUR1 in INS-1 cells. Following docking simulation, the best predicted binding interaction of norbormide (**Figure 6A**) and glibenclamide (**Figure 6B**) with the K_ir_6.1/SUR2 channel showed comparable thermodynamic affinities (−9.2 and −8.7 kcal/mol, respectively), while those towards the K_ir_6.2/SUR1 channel were different (−6.7 and −8.9 kcal/mol; **Figure 6C and D**). Protein–ligand interaction profiler (PLIP) tool results showed interaction network profiles with remarkable differences in the number and bond typologies, as reported in **Figure 6** and **Table 1**.

**Table 1:** Glibenclamide– and norbormide–SUR subunit complex interaction network.

	Hydrophobic interaction	H-bond	π-stacking	π-cation
GLB–SUR1	Ile-381, Trp-430, Thr-588, Leu-592	Tyr-377 Thr-1242 Arg-1246		
NRB–SUR1	Tyr-377, Ile-381, Trp-430, Leu-434	Arg-1300		
GLB–SUR2	Tyr-370, Thr-373, Trp-423, Phe-426, Thr-580, Phe-591, Tyr-1205, Leu-1208	Arg-304 Arg-1213		
NRB–SUR2	Tyr-347, Leu-427, Leu-584, Thr-587, Phe-591	Thr-373 R-1240	Trp-423 Tyr-1205	Arg-1240

Residues engaged in different types of binding interactions were calculated by PLIP tool. NRB: ENDO- and ESO-norbormide.

**Figure 7:**
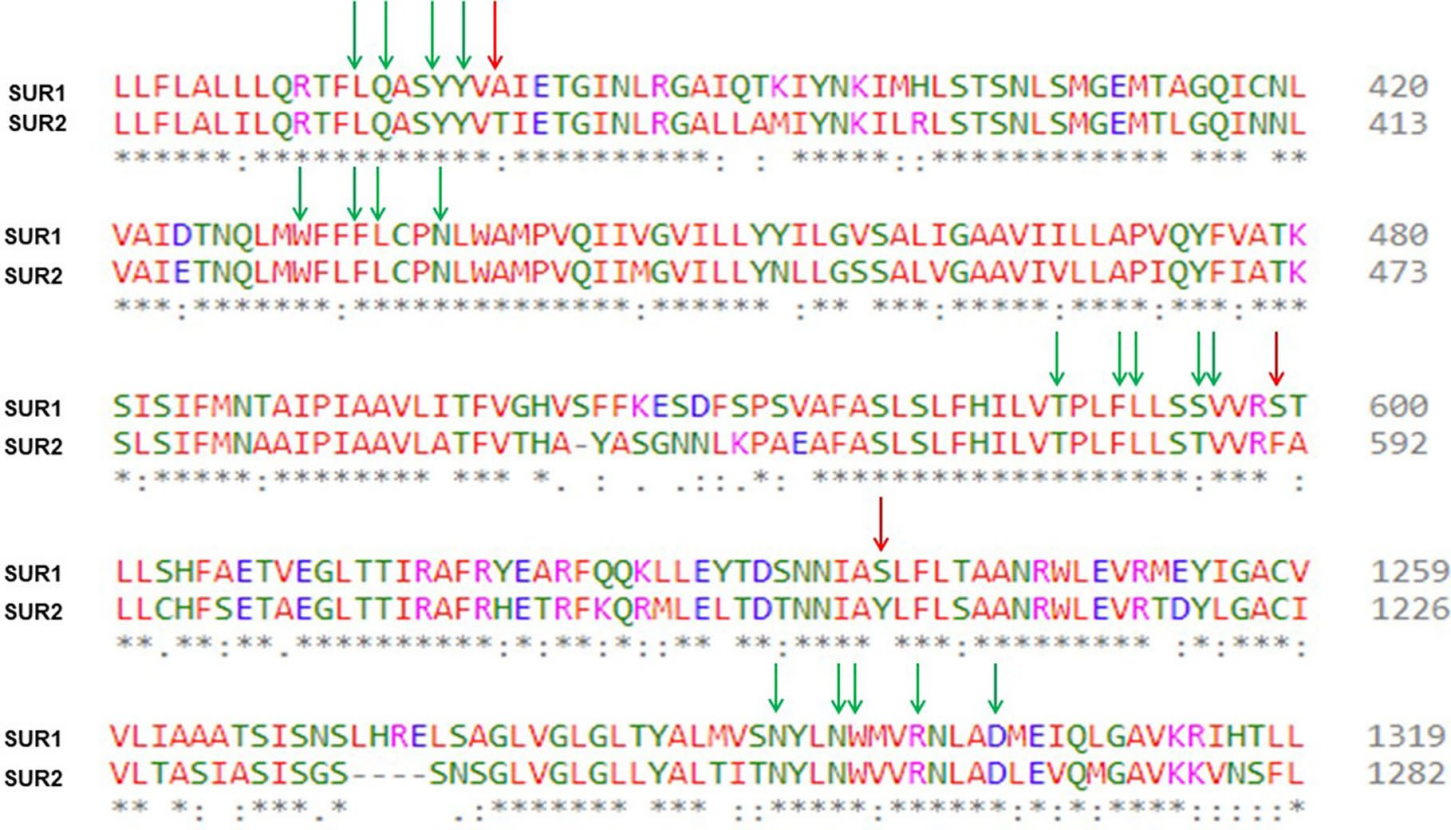
Sequence alignment results between *R. norvegicus* SUR2 and the template sequence of *R. norvegicus* SUR1. Identical and positively conserved amino acids (marked with a star and colon, respectively) are highlighted in colors according to the property of the corresponding query residue in the alignment. Green arrows highlight all the amino acids involved in glibenclamide and norbormide binding regions. Red arrows: non-conserved amino acids engaged in interaction network.

Structural analysis of SUR2–norbormide complex binding pocket residues revealed hydrophobic interactions, π-cationic, π-stacking, and hydrogen bonds, whereas, in the case of the SUR1–norbormide complex, the different amino acid composition prevented analogous interactions, as shown in **Figure 7**. In contrast, glibenclamide formed bonds, similar per number and typologies, with both SUR subunits, as shown in **Figure 6** and **Table 1**.

## Discussion

A key finding of this study is that norbormide inhibits K_ATP_ current in myocytes freshly isolated from the rat caudal artery. This effect was concentration-dependent and was observed at norbormide concentrations overlapping those eliciting a slowly reversible, contractile effect in the rat caudal artery rings (see also Bova et al., 1996).

Pinacidil-induced K_ATP_ current was only partially antagonized by 5 µM norbormide and almost completely blocked by 10 times higher concentrations of the compound. Accordingly, pinacidil failed to counteract the contraction induced by 50 µM norbormide and partly reverted that elicited by a 10 times lower concentration of the compound. Though these data point to K_ATP_ channel blockade as the mechanism underlying norbormide-induced vasoconstriction, other observations presented in this study suggest that this activity is only partially responsible for norbormide vasotonic effect and that there are likely other mechanisms involved. In fact, norbormide-induced K_ATP_ current inhibition could be observed not only with endo-specific norbormide (vasoactive), but also with exo-specific norbormide, known to be inactive as a vasoconstrictor and non-toxic to rats (Poos et al., 1966). Furthermore, K_ATP_ current inhibition could also be demonstrated in myocytes freshly isolated from mouse caudal artery and from rat stomach, i.e., from smooth muscles that are not contracted, but rather relaxed, by norbormide (Bova et al., 2003). Taken together, these findings indicate that the norbormide-induced block of K_ATP_ channels is not sufficient to trigger the contractile process.

Glibenclamide inhibited K_ATP_ current in all the smooth muscle cells investigated, though it was unable to induce contraction in the corresponding whole tissues, i.e., rat caudal artery rings and fundus strips, even at concentrations much higher than those required to block K_ATP_ current. However, not all the arteries are insensitive to glibenclamide: several reports show that this drug causes constriction of coronary arteries in various animal species, including rat (Bril et al., 1992; Imamura et al., 1992; Jackson et al., 1993). In this regard, it is worth noting that norbormide is also endowed with a strong coronaro-constrictor activity, which is observed only in rats (Roszkowski, 1965).

On the one hand, the results of the present study suggest that glibenclamide and norbormide share several functional and electrophysiological properties in vascular and non-vascular smooth muscles. Previous studies also revealed other similarities between the two drugs, i.e., activation of the mitochondrial permeability transition pore (Ricchelli et al., 2005; Skalska et al., 2005; Zulian et al., 2011) and inhibition of Ca_V_1.2 channels (Bian and Hermsmeyer, 1994; Fusi et al., 2002). The latter is fascinating since it apparently contrasts to the blockade of K_ATP_ channels (usually causing membrane potential to depolarize and Ca_V_1.2 channels to open) and the corresponding vasoconstrictor activity. On the other hand, norbormide and glibenclamide differ, as the former did not affect K_ATP_ channel currents of INS-1 cells. This observation suggests a possible selectivity of norbormide towards smooth muscle K_ATP_ channels and indicates that channel inhibition operated by norbormide and glibenclamide is driven by a different mechanism of action. This hypothesis is further supported by the docking results.

The structural analysis of the protein–ligand complex was initially focused on a sequence-conserved, cytoplasmic, physical gate site called G loop (Hibino et al., 2010) that plays a crucial role in the regulation of K_ATP_ gating kinetics (Shimomura et al., 2009; Li et al., 2016; Lu et al., 2016). Docking simulation of norbormide against either K_ir_6.1 or K_ir_6.2 G loop revealed that the compound was unable to dock these binding pockets, owing to its steric hindrance. Instead, a blind docking simulation indicated that norbormide bound K_ATP_ channel in the same pocket of glibenclamide (Martin et al., 2017), though with different interaction profiles and binding energy affinities, which seem to be crucial for their different mechanism of action.

Blockade of K_ATP_ channels, mediated by PKC activation, characterizes the activity of several vasoconstrictors (Bonev and Nelson, 1996; Liu and Khalil, 2018). Noticeably, norbormide-induced contraction of rat caudal artery is accompanied by the activation of the PLC–PKC pathway (Bova et al., 2001). However, neither mouse caudal artery nor rat vascular and gastric smooth muscle was contracted by exo-specific norbormide, although it is capable to block K_ATP_ channels. Therefore, the involvement of PKC in K_ATP_ channel current inhibition operated by norbormide and its isomers can be ruled out.

In conclusion, the selective rat toxicant norbormide is a K_ATP_ channel blocker likely exerting a direct effect on the channel protein. This activity can, at least in part, underline the vasoconstrictor effect observed in the rat tail artery. In addition, the lack of effect of norbormide on K_ATP_ channels in INS-1 cells may suggest a potential selectivity of this compound towards smooth muscle cell channels.

## Data Availabilty Statement

All datasets generated for this study are included in the manuscript and/or the supplementary files.

## Ethics Statement

All animal care and experimental protocols conformed to the European Union Guidelines for the Care and the Use of Laboratory Animals (European Union Directive 2010/63/EU), and had been approved by the Italian Department of Health (666/2015-PR, 650/2015, 41451.N.ZRS/2018).

## Author Contributions

SB, SS, and FF conceived the study and performed the experiments. MB, DR, and BH synthesized norbormide isomers. OS and AT performed the *in silico* analysis. SB, SS, FF, OS, and AT analyzed and interpreted the data. SB, SS, DR, and BH wrote the manuscript. All authors approved the final version of the manuscript.

## Funding

This project was supported by the New Zealand Ministry of Business, Innovation and Employment’s Endeavor Fund C09X1710 (BH, SB, MB, and DR) and by the University of Padua, project no. 148125/14 (SB).

## Conflict of Interest Statement

The authors declare that the research was conducted in the absence of any commercial or financial relationships that could be construed as a potential conflict of interest.
