# Supplementary material for: The Selective Rat Toxicant Norbormide Blocks KATP Channels in Smooth Muscle Cells But Not in Insulin-Secreting Cells
Source: Front Pharmacol. 2019 May 23;10:598. doi: 10.3389/fphar.2019.00598 (PMC6540933; doi:10.3389/fphar.2019.00598)
Supplement: Supplementary file 1 [file Image_1.pdf]

Supplementary Figure 1

^{1}H NMR spectrum of *exo*-norbormide (400 MHz, $CDCl_3$)

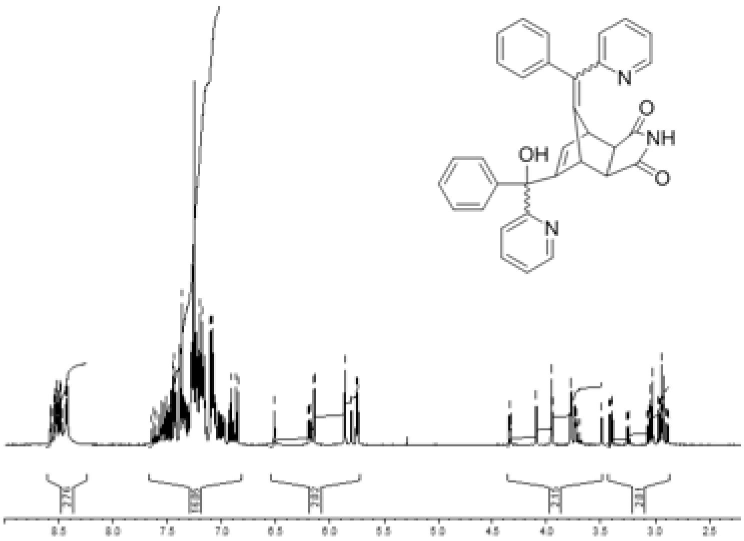

RP-HPLC chromatogram of *exo*-norbormide

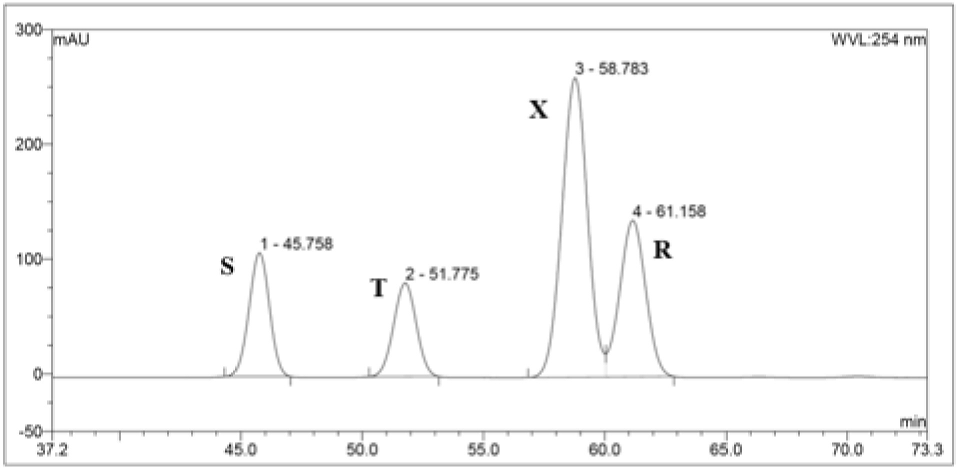

Stereoisomeric composition (%)							
**W**	**S**	**U**	**T**	**X**	**R**	**V**	**Y**
0	**14**	0	**14**	**46**	**26**	0	0
